# Evaluation of headache service quality indicators: pilot implementation in two specialist-care centres

**DOI:** 10.1186/s10194-015-0537-1

**Published:** 2015-06-09

**Authors:** Zaza Katsarava, Raquel Gil Gouveia, Rigmor Jensen, Charly Gaul, Sara Schramm, Anja Schoppe, Timothy J Steiner

**Affiliations:** Department of Neurology, Evangelical Hospital Unna, Holbeinstrasse 10, Unna, 59423 Germany; Department of Neurology, Western German Headache Centre, University of Duisburg-Essen, Essen, Germany; Hospital da Luz Headache Centre, Neurology Department, Hospital da Luz, Lisbon, Portugal; Danish Headache Centre, Department of Neurology, University of Copenhagen, Glostrup Hospital, Copenhagen, Denmark; Migraine and Headache Clinic, Königstein, Germany; Institute of Epidemiology, Biometry and Statistics, University of Duisburg-Essen, Essen, Germany; Department of Neuroscience, Norwegian University of Science and Technology, Trondheim, Norway; Division of Brain Sciences, Imperial College London, London, UK

**Keywords:** Headache disorders, Headache care, Service quality evaluation, Global campaign against headache

## Abstract

**Background:**

Evaluating quality of health care is increasingly recognized as an important contributor to the advancement of health-care delivery. We recently developed a set of quality indicators for headache care, intended to be applicable across countries, cultures and settings so that deficiencies in headache care worldwide might be recognized and rectified. These indicators themselves require evaluation and proof of fitness for purpose. This pilot study begins this process.

**Methods:**

We tested the quality indicators in the tertiary headache centres of the University of Duisburg-Essen in Essen, Germany, and the Hospital da Luz in Lisbon, Portugal. Using seven previously-developed enquiry instruments, we interrogated health-care providers (HCPs), including doctors, nurses, psychologists and physiotherapists, as well as consecutive patients and their medical records.

**Results:**

The questionnaires were easily understood by both HCPs and patients and were not unduly time-consuming. The results from the two headache centres were comparable despite their differences in structure, staffing and language. These findings met the purpose of the study.

Diagnoses were made according to ICHD criteria and critically evaluated during follow-up. However, diagnostic diaries and instruments assessing burden and response to treatment were not always in place or routinely utilised. Triage systems adjusted waiting times to urgency of need. Treatment plans included pathways to other specialities. Patients felt welcomed, reassured and educated, and were mostly satisfied. Discussion points arose over inclusion of psychological therapies in treatment plans; over recording of outcomes; over indicators of efficiency and equitability (protocols to limit wastage of resources, systems to measure input costs and means of ensuring equal access to the services); and over protocols for reporting serious adverse events.

**Conclusion:**

This pilot study to assess feasibility of the methods and acceptability of the instruments of headache service quality evaluation was successful. The project is ready to be taken into its next stages.

## Background

As a feature of health care, quality is obviously desirable. Evaluating quality of health care is increasingly recognized as a necessary link in the advancement of health-care delivery. Yet it is not always clear what “quality” is in this context, and how it is achieved or whether it has been. With regard to headache care, quality has not–until very recently–been defined; quality indicators for headache care that have been developed in the past, in the absence of any agreed meaning of “quality”, are limited to diagnosis and treatment in specific health-care settings, or to specific types of headache.

To address this, so that deficiencies in headache care worldwide might be recognized and rectified, the Global Campaign against Headache [1, 2] in collaboration with the European Headache Federation (EHF) brought together a service quality evaluation (SQE) group of health-services researchers and headache specialists. Their first completed task, after a literature review [3], was to develop a definition of quality, which they conceived as residing in nine separate domains (see Table 1):

**Table 1:** The nine domains of quality in a headache service (4)

Domain A: diagnostic accuracy, therefore asking whether diagnosis were made according to the IHS criteria, documented during the first visit and reviewed during he follow-ups and supported by the diagnostic diaries.
Domain B: issues of the individualized management including waiting time, use of diaries and instruments of headache related disability in treatment plans.
Domain C: availability and utilization of urgent and specialist referral pathways.
Domain D: patient’s education and reassurance
Domain E: convenience, comfort and welcoming of the clinic
Domain F: patient’s satisfaction
Domain G: equity and efficiency of the headache care including access to care, wastage of resources, rate of technical investigations and costs.
Domain H: outcome measures including clinical parameters but also measures of disability and quality of life.
Domain I: safety of care

“Good quality headache care achieves accurate diagnosis and individualized management, has appropriate referral pathways, educates patients about their headaches and their management, is convenient and comfortable, satisfies patients, is efficient and equitable, assesses outcomes and is safe” [4].

Quality evaluation would require application of sets of quality indicators for each of these nine domains separately, and formulation of these was the group’s second completed task [4]. They developed 30 in all, along with related assessment instruments, designed to be applicable across countries, cultures and settings.

Implementation requires the deployment of all of these, at various levels within health systems, most particularly in primary care, which is where most headache should be managed [5, 6]. But prior to this, a number of evaluation studies are needed–in multiple settings, countries and cultures, to demonstrate acceptability of the instruments, with low barriers to usage, and fitness for purpose. Evaluation will require interrogation of health-care providers (HCPs), including doctors, nurses, psychologists and physiotherapists, and of patients, which is not otherwise part of implementation.

Here we report the pilot evaluation study, undertaken in two specialist centres in Europe primarily to learn whether such an enquiry is feasible, and whether the questionnaires are easily understood by both HCPs and patients and not unduly time-consuming. The study continues the collaborative project (headache service quality evaluation) between EHF and *Lifting The Burden* (LTB) within the Global Campaign against Headache [1, 2], which is conducted by LTB in official relations with the World Health Organization.

## Methods

The study was approved by the ethics committees of the University of Duisburg-Essen and Hospital da Luz. Informed consent was obtained from all study participants.

### Study settings and subjects

The headache clinic of the University of Duisburg-Essen is a tertiary headache centre established within a major teaching hospital in Essen, Germany. Medical care is delivered as outpatient or day-clinic care by one senior physician, two residents, two psychologists and one physiotherapist supported by two nurses/secretaries. The centre is supported as necessary by other specialties within the hospital.

The headache clinic of the Hospital da Luz is a specialized centre within a private hospital in Lisbon, Portugal. Medical care includes a daily outpatient clinic, specialist support to the emergency department and inpatient care. One neurology consultant coordinates the centre, which works with physicians from multiple other specialities (physical and rehabilitation medicine, psychiatry, dentistry, gynaecology and obstetrics) and with other HCPs from the hospital, including psychologists, physiotherapists and nurses. The centre has one part-time secretary who coordinates care with the clinical secretaries of each speciality.

In each centre we interviewed the HCPs most involved in delivering the service, and studied consecutive patients and their records. Numbers of patients at each centre were determined by what were feasible.

### Instruments

There were five instruments in total. Among these were three questionnaires: one each for doctors, other HCPs and patients. The last took the form of an exit questionnaire, which patients were asked to complete and return within 2 weeks. In addition, some items of information were extracted from the patients’ records and from central service records.

Table 2 provides an overview of the methods and instruments used. The indicators studied (column one) are grouped in their domains A to I as defined by the SQE group [4]. Column three (Means of enquiry) indicates the method of enquiry: e.g., by questionnaire or chart review. Column two (Measure) explains how each was assessed (many as “yes”/”no”, some quantitatively as continuous measures).

**Table 2:** Methods and outcomes of implementation of quality indicators in each centre

Indicator		Measure	Means of enquiry	Evaluators by centre (percentages are of positive responses)	
				Essen	Lisbon
Domain A. Accurate diagnosis is essential for optimal headache care					
A1	Patients are asked about the temporal profile of their headaches	a) Duration of presenting complaint is recorded in patient’s record (yes/no)	patients’ records	99 %	100 %
A2	Diagnosis is according to current ICHD criteria	a) Diagnosis is recorded in patient’s record (yes/no)	patients’ records	100 %	100 %
		b) Diagnostic record uses ICHD terminology (yes/no)	patients’ records	100 %	92 %
A3	A working diagnosis is made at the first visit	Working diagnosis at first visit is recorded in patient’s record (yes/no)	patients’ records	100 %	92 %
A4	A definitive diagnosis is made at first or subsequent visit	Definitive diagnosis is recorded in patient’s record or, if not, an appointment for review has been given (yes/no)	patients’ records	98 %	92 %
A5	Diagnosis is reviewed during later follow-up	Diagnostic review during follow-up is routinely undertaken (yes/no)	doctors’ questionnaire	100 %	100 %
A6	Diaries are used to support or confirm diagnosis	The service has a diagnostic diary available, and doctors are aware of its availability (yes/no)	doctors’ questionnaire	100 %	100 %
Domain B. Individualized management is essential for optimal headache care					
B1	Waiting-list times for appointments are related to urgency of need	a) Waiting-list times are recorded in database (yes/no)	patients’ records	0 %	0 %
		b) A formal triage system exists to expedite appointments in cases of perceived urgency (yes/no)	doctors’ questionnaire	yes	yes
B2	Sufficient time is allocated to each visit for the purpose of good management	a) Actual time (minutes) per visit is recorded by patient in exit questionnaire: 1st visits	patients’ questionnaire	46 ± 30	25 ± 7
		follow up visits		27 ± 30	24 ± 9
		b) Patient is satisfied^a^ with actual time (yes/not yes)	patients’ questionnaire	100 %	92 %
		c) Health-care providers express overall satisfaction (yes/no)	patients’ questionnaire	83 %	100 %
			doctors’ and other HCPs’ questionnaires		
B3	Patients are asked about the temporal profile of their headaches	Frequency (or days/month) of symptoms is recorded in patient’s record (yes/no)	patients’ records	100 %	100 %
B4	Treatment plans follow evidence-based guidelines, reflecting diagnosis	Prescribed drugs (names, doses and quantities) are recorded in patient’s record	patients’ records	100 %	96 %
B5	Treatment plans include psychological approaches to therapy when appropriate	a) Access route to psychological therapies exists (yes/no)	doctors’ questionnaire	yes	yes
		b) Utilisation is recorded in patient’s record	patients’ records	100 %	32 %
B6	Treatment plans reflect disability assessment	a) An instrument for disability assessment is available (yes/no) and is appropriate in the setting (yes/no)	doctors’ questionnaire	yes	yes
				yes	yes
		b) Disability is recorded in patient’s record (yes/no)	patients’ records	0 %	100 %
B7	Patients are followed up to ascertain optimal outcome	a) Follow-up appointment dates appear in central service records	central service records	36 %	32 %
		b) A follow-up diary and/or calendar is available (yes/no)	doctors’ questionnaire	yes	yes
Domain C. Appropriate referral pathways are essential for optimal headache care					
C1	Referral pathway is available from primary to specialist care	A usable pathway exists (yes/no)	doctors’ questionnaire	yes	yes
C2	Urgent referral pathway is available when necessary	A usable pathway exists (yes/no)	doctors’ questionnaire	yes	yes
Domain D. Education of patients about their headaches and their management is essential for optimal headache care					
D1	Patients are given the information they need to understand their headache and its management	Patient is satisfied^a^ with information given (yes/not yes)	patients’ questionnaire	99 %	92 %
D2	Patients are given appropriate reassurance	Patient is satisfied^a^ with reassurance given (yes/not yes)	patients’ questionnaire	100 %	94 %
Domain E. Convenience and comfort are part of optimal headache care					
E1	The service environment is clean and comfortable	a) Patient is satisfied^a^ with cleanliness and comfort (yes/not yes)	patients’ questionnaire	98 %	94 %
		b) Health-care providers are satisfied with cleanliness and comfort (yes/no)	doctors’ and other HCPs’ questionnaires	67 %	60 %
E2	The service is welcoming	Patient is satisfied^a^ with welcome (yes/not yes)	patients’ questionnaire	100 %	94 %
E3	Waiting times in the clinic are acceptable	a) Waiting time (minutes) is recorded by patient in exit questionnaire	patients’ questionnaire	20 ± 18	23 ± 23
		b) Patient is satisfied^a^ with waiting time (yes/not yes)	patients’ questionnaire	88 %	78 %
		c) Health-care providers are satisfied with waiting times (yes/no)	doctors’ and other HCPs’ questionnaires	100 %	60 %
Domain F. Achieving patient satisfaction is part of optimal headache care					
F1	Patients are satisfied with their management	Patient is satisfied^a^ with overall management (yes/not yes)	patients’ questionnaire	96 %	74 %
Domain G. Optimal headache care is efficient and equitable					
G1	Procedures are followed to ensure resources are not wasted	A protocol to limit wastage exists (yes/no)	doctors’ questionnaire	no	no
G2	Costs of the service are measured as part of a cost-effectiveness policy	A record of input costs exists (yes/no)	doctors’ questionnaire	yes	yes
G3	There is equal access to headache services for all who need it	A policy to ensure equal access exists (yes/no)	doctors’ questionnaire	no	no
Domain H. Outcome assessment is essential in optimal headache care					
H1	Outcome measures are based on self-reported symptom burden (headache frequency, duration and intensity)	a) An outcome measure (HURT or similar) is available (yes/no)	doctors’ questionnaire	yes	yes
		b) Outcomes according to this measure are recorded in patient’s record (yes/no/not applicable)	patients’ records	0 %	68 %
H2	Outcome measures are based on self-reported disability burden	a) An outcome measure (HALT or similar) is available (yes/no)	doctors’ questionnaire	no	yes
		b) Outcomes according to this measure are recorded in patient’s record (yes/no/not applicable)	patients’ records	na	68 %
H3	Outcome measures are based on self-reported quality of life	a) An outcome measure (WHOQoL or similar) is available (yes/no)	doctors’ questionnaire	no	no
		b) Outcomes according to this measure are recorded in patient’s record (yes/no/not applicable)	patients’ records	na	na
Domain I. Optimal headache care is safe					
I1	Patients are not over-treated^b^	Prescribed drugs (names, doses and quantities) are recorded in patient’s record (yes/no/not applicable)	patients’ records	100 %	100 %
I2	Systems are in place to be aware of serious adverse events^c^	a) Serious adverse events are recorded	patients’ records, central service records	none	none
		b) A protocol exists for reporting serious adverse events (yes/no)	doctors’ questionnaire	no	yes

*HCP* Health-care provider, *na* not applicable, *ICHD* International Classification of Headache Disorders, *HURT* Headache Under-Response to Treatment questionnaire [8], *HALT* Headache-Attributed Lost Time questionnaire [10]

^a^ Patient’s satisfaction was elicited either from the options “yes” / ”no”, or as the middle option of “too much” / ”about right” / ”too little”, or as “very good” or “good” on a Likert scale extending through “adequate”, “poor” and “very poor”

^b^ Over-treatmentmay mean excessive use of drugs likely to induce MOH, overdosage with potentially harmful drugs such as ergotamine or steroids, use of prophylactics for infrequent headache, use of prophylactics for the wrong diagnosis, or use of non-evidence-based treatments that are unlikely to be effective \and may jeopardize safety

^c^ Serious adverse events are those that cause death, are life-threatening, terminate or put at risk a pregnancy, or cause hospitalization, prolonged illness, disability and/or malignancy

Questionnaires were translated from their English originals into German and Portuguese by three authors (ZK, CG and RG) according to *Lifting The Burden*’s translation protocol for hybrid documents [7], using forward and backward translation, with colleagues and patients not involved in the study providing review and assistance.

### Data collection

HCPs and patients completed questionnaires anonymously. Data from patients’ records were extracted by a medical student (AS) in Essen and by a clinical research assistant in Lisbon. Information from central service records was supplied by the HCPs.

### Analysis

Demographic and clinical data are provided as numerical values, percentages or mean values with standard deviations (SDs). No statistical tests were performed.

## Results

In Essen we interviewed six HCPs: one senior physician, two residents, two nurses and one psychologist. We interviewed and reviewed the records of 89 patients, of whom 22 were male, 67 female, mean age 42.5 ± 12.7 years. Most (61) had migraine, 53 episodic and eight chronic; 13 had tension-type headache (TTH), 9 episodic and four chronic; 18 had medication-overuse headache (MOH), one had cluster headache (CH) and one had trigeminal neuralgia (TN). Five patients were given two diagnoses.

In Lisbon we interviewed 10 HCPs: one neurologist, one dental surgeon, two psychiatrists, two physiotherapists and four gynaecologists. We interviewed and reviewed the records of 50 patients, five male and 45 female, mean age 37.3 ± 11.2 years. Again, most patients (40) had migraine, seven had TTH, four had MOH and one each had new daily persistent headache, TN and orgasmic cephalalgia. Four patients were given two diagnoses.

On a general level, questionnaires proved easy to apply, were readily understood and accepted by both HCPs and patients, and not unduly time consuming. None of the specific enquiries caused or led to difficulties. Evaluation of each clinic according to the quality indicators is shown in Table 2. Despite the two different languages, German and Portuguese, the different settings and the very different structures of the two centres, the findings were very comparable. We summarise them by domain.

### Domain A: accurate diagnosis

Diagnoses were made according to current ICHD criteria at both centres, documented after the first visit and reviewed after follow-up with the support of diagnostic diaries. Percentages of positive responses were ≥98 % in Essen and ≥92 % in Lisbon (Table 2).

### Domain B: individualized management

A triage system to identify urgent cases existed in both clinics, but waiting-list times until first appointment were not documented. Mean time allocated to patients’ visits (according to patients’ reports) was 46 min for the first visit in Essen (with wide variation), but half that (and with much less variation) in Lisbon. Patient satisfaction (Essen 100 %, Lisbon 92 %) did not appear to be greatly influenced by this (Table 2). Treatment plans followed international guidelines. Lisbon made less use of psychological treatment. Disability measures existed in both clinics; disability was well documented in Lisbon (100 %), but not at all in Essen. In both clinics, only one third of patients were routinely followed to ascertain optimal outcomes (Table 2).

### Domain C: appropriate referral pathways

Both clinics were well established with referral pathways for urgent and specialist consultations (Table 2).

### Domain D: education and reassurance of patients

The great majority of patients expressed satisfaction (Essen ≥99 %; Lisbon ≥92 %) on both counts (Table 2).

### Domain E: convenience and comfort

More patients (94–98 %) than HCPs (60–67 %) found the service environment clean and comfortable; most patients felt welcomed (Essen 100 %, Lisbon 94 %). Waiting times in the clinic varied quite widely around means of 20–23 min, and were unsatisfactory for a sizeable minority of patients (Essen 12 %, Lisbon 22 %) and 40 % of HCPs in Lisbon (Table 2).

### Domain F: patient satisfaction

Overall satisfaction with their management was expressed by most patients (Essen 96 %, Lisbon 74 %) (Table 2).

### Domain G: efficiency and equitability

Neither clinic had a protocol to avoid wastage of resources. Running costs were calculated, but the information was available to senior management only. Neither clinic was able to offer equal access to headache services for all who might need it.

### Domain H: outcome assessment

Outcome measures were available in both clinics (in Essen only for symptom burden, in Lisbon also for disability), but they were used only in Lisbon. Neither clinic evaluated quality of life.

### Domain I: safety

The service was safe in Lisbon, and probably also in Essen. Prescribed drugs were always well documented in both centres. In both, also, there were no recorded serious adverse events (SAEs), but the pilot identified absence in Essen of any formal protocol to ensure that SAEs were reported (Table 2).

## Discussion

This was a pilot study to assess feasibility of the methods and acceptability of the instruments before committing resources to a large multicentre implementation study, which–still in the evaluation phase of the project–must be done next. The pilot was successful in this purpose: the questionnaires were found easy to apply and were readily understood and acceptable, and, importantly, the whole process proved not unduly time consuming. All this was so in both German and Portuguese languages, and in two centres with very different structures–one in a university teaching hospital and one in a private hospital.

Also in these two very different settings, findings with regard to quality were quite comparable. We can summarise these findings, while emphasising that here we were testing the concepts behind the quality evaluation project, *not* the centres, and we were certainly not comparing the centres. Triage systems were in place to adjust waiting times to first appointment to urgency of need. Waiting times to be seen were generally considered acceptable and most patients felt welcomed, reassured and satisfied. Diagnoses were made according to current ICHD criteria, and were critically evaluated during follow-up visits. Treatment plans included psychological therapies (variably utilised) and pathways to other specialities. Outcomes were assessed on symptom measures (such as headache intensity and frequency) and on disability burden in Lisbon. However, instruments to assess outcomes were not always in place, or not routinely utilised.

Both services would be considered to have failed on indicators of efficiency and equitability. Protocols for limiting wastage of resources did not exist; input costs were measured, but the personnel utilising resources were not informed of them; equal access to the services was not ensured.

The services appeared safe, but we say more about this below.

There are two lines of thought worth further discussion. One is to look at these findings and, where problems appeared, question whether these truly reflected issues of *quality*. The other is to identify the next steps in evaluation of the quality indicators.

Pursuing the first of these, we see no issues arising in domain A. In domain B, the centres differed markedly in their utilisation of psychological management approaches (100 % at Essen, 32 % at Lisbon), although both had access. They differed even more in recording disability in patients’ notes in order that management plans might reflect this (0 % at Essen, 100 % at Lisbon). We will come back to whether or not these are issues of quality. Both centres showed evidence of routinely following-up only one third of patients. In primary care, the expectation would be close to 100 %, whereas specialist-care practice entirely appropriately sends many patients back to primary care, with detailed advice for follow-up. Domains C and D raised no issues, interestingly in the latter case since it recorded patient satisfaction, which is notoriously fickle. That fickleness may be evident in domain F: Lisbon may be disappointed with an overall patient satisfaction rating of 74 % in the face of much higher scores elsewhere (although only 78 % for waiting times–domain E). Do patients really attach so much more importance to waiting time than to time spent with the doctor (92 % satisfied), information received (92 %) and reassurance (94 %), cleanliness and comfort (94 %) and a warm welcome (94 %)? When developing these indicators, the SQE group found it impossible to exclude patient satisfaction [4], but it can be difficult to understand what are its determinants. In fact, waiting times were clearly a problem at Lisbon, since the HCPs expressed even less satisfaction with them (60 %), but only to a limited extent are they within the control of clinicians. These quality indicators are equally for service managers, who have responsibility for resource allocation and for protocols avoiding wastage of resources, and who, perhaps, can do something to promote equitable access.

The SQE group did not incorporate clinical outcomes themselves into the quality indicators because they found no objective basis for stipulating what outcomes should be considered optimal in individual cases in particular settings [4]. Instead they called for evidence that outcomes were recorded against recognised outcome measures. Neither centre apparently attached great importance to this: Lisbon made some use of recognised outcome measures in two thirds (68 %) of cases, Essen not in any. Both centres did, nonetheless, record outcomes in terms of symptoms–in particular, headache frequency–in patients’ records. The quality issues related to this are threefold. First, accurate recording of outcomes serves patient follow-up and guides achievement of best outcomes. Second, it is part of good record-keeping, failure of which would be clear evidence of a quality deficiency. However, *recognised* outcome measures are not needed for either of these purposes: any system that has meaning to its users may be sufficient, including simple but careful recordings of symptoms in patients’ notes. But the third is this: how does a service know what its outcomes are; how can it assess itself against benchmarks; how can it improve (or even know whether it needs to improve)? This purpose is quite different, and requires that outcomes are *formally* recorded, in a standard manner, and it is for this purpose that recognised outcome measures are mandatory.

Since no outcome measures are yet universally accepted, it is part of the service quality evaluation project to suggest those that might be used and include them in the evaluations. The HURT questionnaire was developed as an outcome measure specifically for the purpose of achieving best outcomes [8]. While it was intended primarily for non-specialists, it has much broader utility [9], reflected in the fact that both centres had it available. Lisbon made use of it for the majority of their patients. For disability burden, the HALT questionnaire [10], like MIDAS from which it is derived [11], records lost productive time–which correlates strongly with disability. The two instruments have been widely validated, and HALT is available in multiple European translations. As for quality of life, while WHOQoL-8 [12] was suggested, there is no evidence that this is useful as an outcome measure in headache management. Neither is there good support for any alternative. Future studies may conclude that this indicator should not be retained.

Finally, on the matter of safety, the issue again is one of record-keeping. It may be that a protocol for reporting SAEs at Essen had not been required because none had occurred, but managers should have recognised the need to have one in place. It is not a minor point. Essen would have been considered to have failed on this indicator: it could not be known for certain whether there had been no SAEs at Essen or whether they were merely not recorded.

The second line of thought is to consider the next steps in developing the methodology and instrumentation of headache service quality evaluation. Following this pilot, the quality indicators will be implemented in many centres in a Europe-wide study supported by the European Headache Federation and *Lifting The Burden*, still in specialist care. The protocol will be the same, with the seven instruments used to evaluate performance. This extended study will serve two purposes. First it will confirm (or not) that the indicators are fit for purpose, with or without some degree of refinement. Second, by establishing what is majority practice, it will guide the setting of benchmarks against which quality may be judged. Uncertainties, such as the extent to which psychological approaches should be used in management, and whether and to what extent management plans should be based on disability assessments, on which Essen and Lisbon clearly differed, may not belong in indicators of quality. We shall look for consensus in this study. There will be both qualitative and quantitative benchmarks to be set. The danger, of course, is to assume that majority practice correctly sets the benchmark; but that is a matter for these future studies.

The stage after that will be to take the process into non-specialist care–primary care in particular. It is envisaged that the finally-agreed quality indicators should not themselves be varied when taken into primary care, but the benchmarks may be different.

## Conclusion

This pilot study to assess feasibility of the methods and acceptability of the instruments of headache service quality evaluation was successful. The project is ready to be taken into its next stages.

## Abbreviations

CH: Cluster headache
EHF: European Headache Federation
ICHD: International classification of headache disorders
HCP: Health-care practitioner
LTB: *Lifting The Burden*
MOH: Medication-overuse headache
SAE: Serious adverse event
SD: Standard deviation
SQE: Service quality evaluation
TTH: Tension-type headache
TN: Trigeminal neuralgia
